# Sensor-Based Monitoring of Bolted Joint Reliability in Agricultural Machinery: Performance and Environmental Challenges

**DOI:** 10.3390/s25165098

**Published:** 2025-08-16

**Authors:** Xinyang Gu, Bangzhui Wang, Zhong Tang, Haiyang Wang

**Affiliations:** 1 School of Agricultural Engineering, Jiangsu University, Zhenjiang 212013, China; 2 Key Laboratory of Modern Agricultural Equipment and Technology, Ministry of Education, Jiangsu University, Zhenjiang 212013, China

**Keywords:** bolted joint, structural reliability, bolt loosening, condition monitoring, agricultural machinery

## Abstract

The structural reliability of agricultural machinery is critically dependent on bolted joints, with loosening being a significant and prevalent failure mode. Harsh operational environments (intense vibration, impact, corrosion) severely exacerbate loosening risks, compromising machinery performance and safety. Traditional periodic inspections are inadequate for preventing sudden failures induced by loosening, leading to impaired efficiency and safety hazards. This review comprehensively analyzes the unique challenges and opportunities in monitoring bolted joint reliability within agricultural machinery. It covers the following: (1) the status of bolted joint reliability issues (failure modes, impacts, maintenance inadequacies); (2) environmental challenges to joint integrity; (3) evaluation of conventional detection methods; (4) principles and classifications of modern detection technologies (e.g., vibration-based, acoustic, direct measurement, vision-based); and (5) their application status, limitations, and techno-economic hurdles in agriculture. This review identifies significant deficiencies in current technologies for agricultural machinery bolt loosening surveillance, underscoring the pressing need for specialized, dependable, and cost-effective online monitoring systems tailored for agriculture’s demanding conditions. Finally, forward-looking research directions are outlined to enhance the reliability and intelligence of structural monitoring for agricultural machinery.

## 1. Introduction

**Figure 1:**
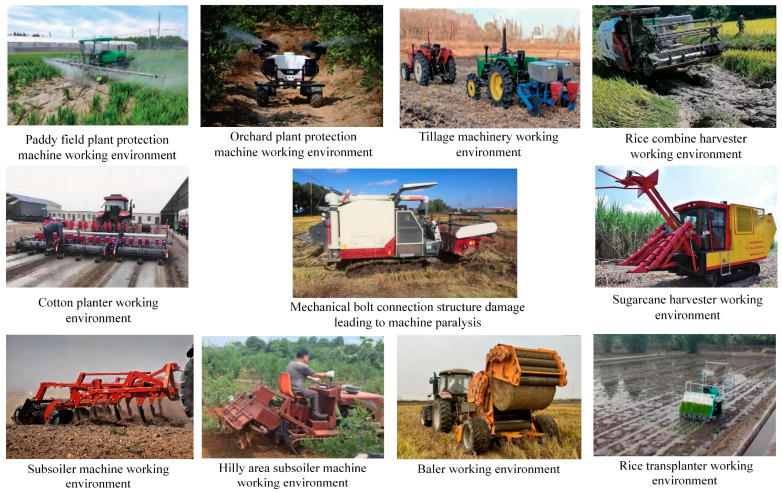
Complex field operational environments and structural failures can cause the complete breakdown of some agricultural machinery.

Modern agriculture is undergoing a profound transformation towards enhanced efficiency, precision, and sustainability, and within this transition, agricultural mechanization and intelligence function as indispensable cornerstones [1,2,3,4]. The deployment of advanced agricultural equipment—such as tractors, combine harvesters, and precision planters—not only substantially increases labor productivity and operational efficiency [5,6] but also provides the technological foundation for modern agricultural paradigms like precision and smart farming [7]. Agriculture is a seasonal activity, and agricultural machinery is machinery that is used seasonally, with some machines only being used for a few days during the year. These machines are exposed to the effects of dust, rain, mud, snow, and other influences. Very often these machines are located outdoors and are not protected from weather conditions throughout the year. These are just some of the reasons that indicate the problem of difficult maintenance of all working elements, especially screw elements [8,9,10,11]. These uniquely harsh working conditions impose stringent demands on structural integrity, functional stability, and long-term reliability [12]. Critically, unplanned downtime resulting from mechanical or structural failures during peak agricultural seasons can precipitate substantial economic losses and crop yield reductions [13,14]. Therefore, ensuring and enhancing the reliability of agricultural machinery represent a central theme in modern agricultural engineering, vital for propelling the agricultural modernization process [15]. Within the large-scale and intricate structural framework of agricultural machinery (Figure 1), bolted connections represent the most prevalent and fundamental means for component fixation and assembly, owing to inherent advantages such as standardization, ease of assembly/disassembly, and cost-effectiveness [16,17]. Their application is widespread, spanning the attachment of power systems (engines, transmissions) to frames, the assembly of load-bearing structures like chassis, suspension systems, and running gear, and the securing of various direct soil-engaging or crop-processing components (e.g., plowshares, rotary blades, harvester header elements) [18,19]. A single large agricultural machine often incorporates thousands of such connection points, which function critically to integrate individual parts into a cohesive, operational whole, responsible for load transmission, maintenance of geometric precision, and ensuring structural rigidity and strength [20,21]. Consequently, the tightening status and overall health of these numerous bolted joints directly govern the machine’s operational performance, work quality, service life, and, ultimately, operational safety [22].

**Figure 2:**
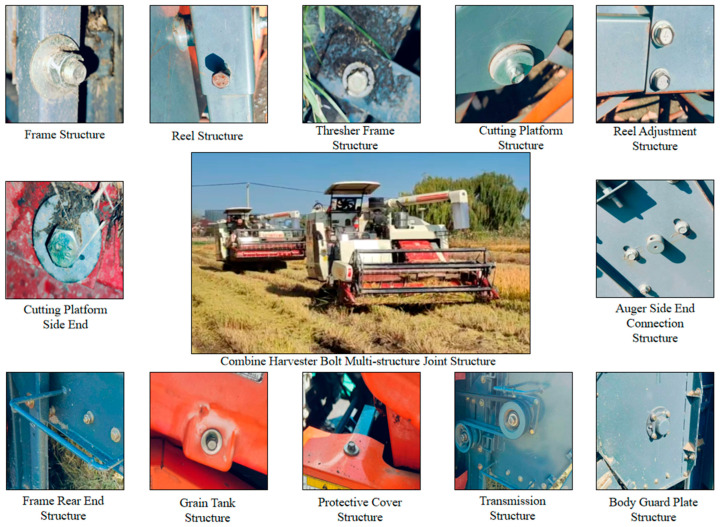
Bolted connections are employed for assembling the complex structures of agricultural machinery.

The reliability of bolted connections fundamentally underpins the overall dependability of agricultural machinery. Taking the combine harvester as an example, a complex agricultural machine utilizing numerous bolted structures as illustrated in Figure 2, continuous operating vibrations caused primarily by the complex operating conditions of agricultural machinery, primarily movement on uneven terrain, act as a primary external driver, causing micro-movement and rotational tendencies in the bolts, culminating in self-loosening. Despite their indispensability, bolt loosening persists as a chronic and difficult-to-remedy issue during the practical operation of such machinery [23]. Continuous operational vibration acts as the primary external driver, inducing micro-motion and rotational tendencies in bolts, which culminates in self-loosening [24,25]. Concurrently, factors including material creep, stress relaxation, settlement at the connection interfaces, and environmental corrosion contribute synergistically to the gradual decay and loss of bolt preload [26,27]. The consequences of bolt loosening or inadequate preload are significant, i.e., the initiation of abnormal component vibration and noise [28], reduction in connection strength and stiffness, acceleration of fatigue damage accumulation [29], and potentially progressing to complete joint failure and component detachment, thereby precipitating severe mechanical failures or safety incidents [30].

Current maintenance protocols for bolted connections, prevalent in both agricultural machinery and other industrial sectors, largely depend on operator experience and fixed-interval inspections involving torque re-application. Such approaches are inherently inefficient, impeding timely intervention during the initial stages of loosening or significant preload reduction [31,32]. This traditional methodology starkly conflicts with the requirements of modern agricultural equipment for enhanced reliability, predictive maintenance strategies, and intelligent management systems [33]. Consequently, the development of advanced monitoring technologies is urgently necessitated to enable real-time, online, and accurate assessment of critical bolted joint conditions in agricultural machinery, with a particular emphasis on early warning capabilities for loosening faults [34,35].

Addressing the existing bottlenecks in monitoring bolted connection reliability within the agricultural machinery context, this paper synthesizes research advancements across five critical domains to propose technological pathways for overcoming these challenges: (1) the current status of bolted joint reliability problems in agricultural machinery, encompassing typical failure modes, impacts on overall machine performance, and deficiencies in existing maintenance management; (2) the challenges posed by the unique operational conditions and environments of agricultural machinery to both joint reliability and the effective implementation of monitoring technologies; (3) an evaluation of the principles, characteristics, and practical effectiveness of conventional bolt loosening detection methods employed in agricultural machinery maintenance; (4) the fundamental principles and classifications of modern bolt loosening detection technologies, including those based on distinct sensing modalities such as vibration, acoustics, direct measurement, and vision; and (5) the specific application status, performance assessments, principal limitations, and prevailing technical and economic bottlenecks associated with the deployment of these various modern detection techniques within the agricultural machinery domain. This research was initiated through a comprehensive literature review, retrieving pertinent publications from the Web of Science database and the China National Knowledge Infrastructure (CNKI), resulting in the review of 185 articles.

## 2. Current Status of Reliability Issues in Agricultural Machinery Bolted Connections

The operational reliability of agricultural machinery directly dictates agricultural production efficiency and effectiveness. As the most fundamental and extensively employed structural joining method, the reliability status of bolted connections constitutes a critical cornerstone for overall machine dependability [36]. However, within the complex and severe operational environments characteristic of agricultural applications, bolted connection reliability problems, particularly loosening failures, have emerged as a pervasive and pressing challenge requiring urgent attention [37,38]. This section provides a detailed exposition of the typical bolt loosening failure modes encountered during agricultural machinery operation, delineates the specific detrimental impacts of loosening on machine performance and operational efficiency, and critically examines the inadequacies inherent in current maintenance paradigms regarding the effective management of bolted connections.

### 2.1. Typical Bolt Loosening Failure Modes During Agricultural Machinery Operation

**Figure 3:**
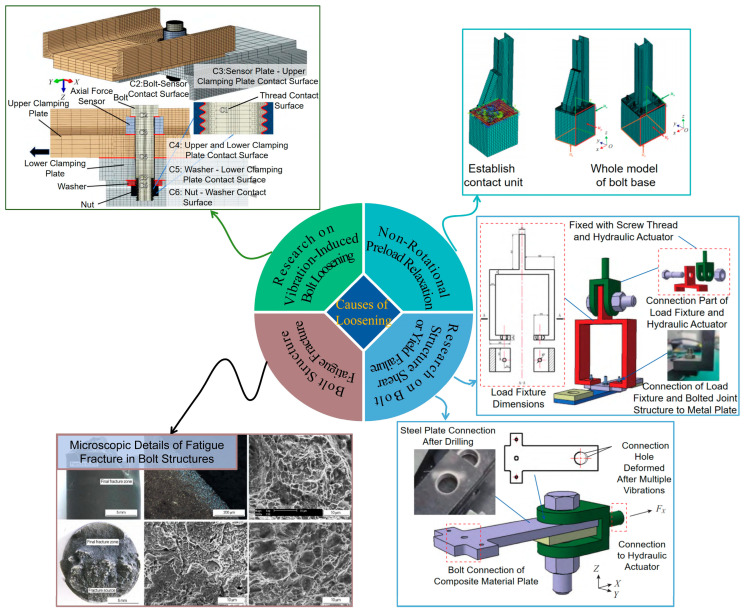
Typical failure modes of bolts.

During field operations, agricultural machinery is subjected to intense vibrations and impact loads from multiple sources, including uneven ground surfaces, engine operation, collisions between working components [39], and the transmission of power through universal joints. These factors can compromise the overall reliability of bolted mechanical structures and their screw components [40]. This complex dynamic loading environment constitutes the primary external factor inducing loosening failure in bolted connections. Typical failure modes of bolted connection structure, as illustrated in Figure 3, primarily include the following:

Vibration-induced self-loosening: This represents the most prevalent and extensively studied loosening mode [41]. Under the influence of transverse vibration (perpendicular to the bolt axis), minute relative slip occurs at the contact interfaces of the thread pair and between the nut/bolt head and the clamped components [42,43]. When the vibration amplitude is sufficiently large, this micro-slip overcomes the frictional forces, leading to gradual reverse rotation of the nut (or bolt), thereby causing a rapid decrease in bolt preload [44,45]. While axial vibration typically does not directly induce rotation, it causes periodic fluctuations in preload, which can also exacerbate the loosening process [46]. Many components on agricultural machinery, such as engine mounts, transmission connections, and suspension systems, are persistently exposed to this high-risk vibratory environment [47].

Non-rotational preload relaxation: Even in the absence of bolt rotation, preload can diminish gradually due to other mechanisms [48], including the following: (1) Embedding loss: Under high preload, micro-asperities on the contact surfaces (threads, under-head/nut faces, clamped component surfaces) are flattened through localized plastic deformation. This results in a reduction in the effective bolt elongation, leading to a decrease in preload [49,50]. (2) Creep and stress relaxation: exposure to sustained loads and/or elevated temperatures (e.g., for bolts located near engines or exhaust systems) can induce creep or stress relaxation in the bolt material or the clamped components, causing a time-dependent reduction in preload [51,52]. (3) Gasket relaxation: if sealing gaskets are incorporated within the connection, preload loss can also occur due to the compression set or aging of the gasket material [53].

Fatigue fracture: Bolted connections are susceptible to fatigue fracture under the influence of alternating loads, particularly in regions of high stress concentration such as thread roots and the head-to-shank fillet transition [54,55]. In the context of uneven tightening forces in group threaded joints, the best option is the synchronous tightening of threaded joints using multi-spindle high-precision equipment [56]. A reduction in preload consequent to loosening significantly elevates the amplitude of alternating stress experienced by the bolt. This dramatically curtails the bolt’s fatigue life, accelerating the initiation and propagation of fatigue cracks, ultimately culminating in sudden bolt fracture [57]. For critical connections subjected to dynamic loading on agricultural machinery, including connecting rod bolts and wheel hub bolts, the risk of fatigue fracture following loosening is exceptionally high [58].

Shear or yield failure: Bolted connections primarily depend on the friction generated by preload to counteract transverse loads. When preload becomes severely inadequate due to loosening, the transverse load must be resisted predominantly by the shear strength of the bolt shank [59]. If the magnitude of this transverse load surpasses the bolt’s capacity, it can lead to either shear fracture or plastic yielding of the bolt material, resulting in the complete failure of the connection [60].

These failure modes rarely manifest in isolation; instead, under the complex operational conditions of agricultural machinery, they often interact and mutually exacerbate, synergistically contributing to the degradation of bolted connection reliability.

### 2.2. Impacts of Bolt Loosening on Agricultural Machinery Performance and Operational Efficiency

**Figure 4:**
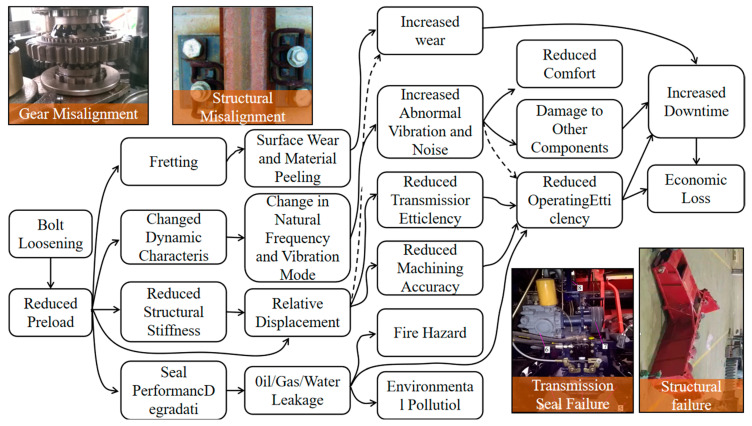
Detrimental effects of bolt loosening on combine harvesters.

Bolt loosening represents more than localized connection failure; its detrimental effects propagate throughout the affected component and potentially the entire machine system, as illustrated in Figure 4, negatively influencing multiple aspects of agricultural machinery performance and operational efficiency:

Reduction in structural stiffness and alteration in dynamic characteristics: Diminished bolt preload directly results in a consequent reduction in joint stiffness [61]. This alteration subsequently modifies the natural frequencies and vibrational modes of the component or the entire machine, potentially inducing unintended resonance phenomena. Such resonance can lead to abnormal vibrations and increased noise levels, compromising operator comfort and potentially accelerating the degradation of other components.

Inducement of component misalignment and loss of precision: For assemblies requiring precise positioning, such as gearboxes, bearing housings, and cutting units, bolt loosening precipitates relative displacement and misalignment between components. This consequently leads to diminished transmission efficiency, accelerated wear, and compromised operational accuracy.

Inducement of leakage problems: In connections requiring a seal, such as hydraulic system joints, engine cylinder heads, and oil pans, insufficient preload compromises sealing performance, leading to the leakage of oil, gas, or water. This not only results in the wastage of energy or operational media but also poses risks of environmental contamination and potentially triggers safety incidents like fires.

Reduction in operational efficiency and increased downtime: The aforementioned performance issues directly diminish the field operational efficiency of agricultural machinery. More critically, bolt loosening frequently culminates in component damage or connection failure, necessitating unscheduled downtime for repairs. Such unplanned interruptions to operations invariably lead to significant economic losses [62,63].

### 2.3. Deficiencies in Bolted Connection Management Under Existing Maintenance Paradigms

Currently, maintenance management strategies for bolted connections on agricultural machinery predominantly rely on traditional approaches, such as operator experience-based assessments or predetermined fixed-interval schedules. While these methods are widely practiced, they inherently face the following challenges in ensuring optimal reliability due to the varying and dynamic operating conditions:

(1) Low reliability: manual checks are subjective and often fail to detect early or minor loosening/preload decay. (2) Torque inaccuracy: periodic re-torquing [64] is unreliable as applied torque poorly correlates with actual preload due to variable factors (e.g., friction), risking incorrect clamping force and offering no insight into status changes between intervals. (3) Lack of predictive capability: these reactive or scheduled approaches [65] lack real-time condition data, hindering predictive maintenance and leading to potential under- or over-maintenance. (4) Inefficiency and accessibility issues: poor accessibility of bolts on compact machinery makes inspections difficult, time-consuming, and costly.

In summary, the prevailing maintenance paradigms are insufficiently equipped to effectively manage the pervasive risks associated with bolt loosening in agricultural machinery. This situation highlights an urgent requirement for the integration of more advanced, dependable, and efficient monitoring technologies and management strategies.

## 3. Impacts of Specialized Agricultural Machinery Operating Conditions and Environments on Bolted Connection Reliability

**Figure 5:**
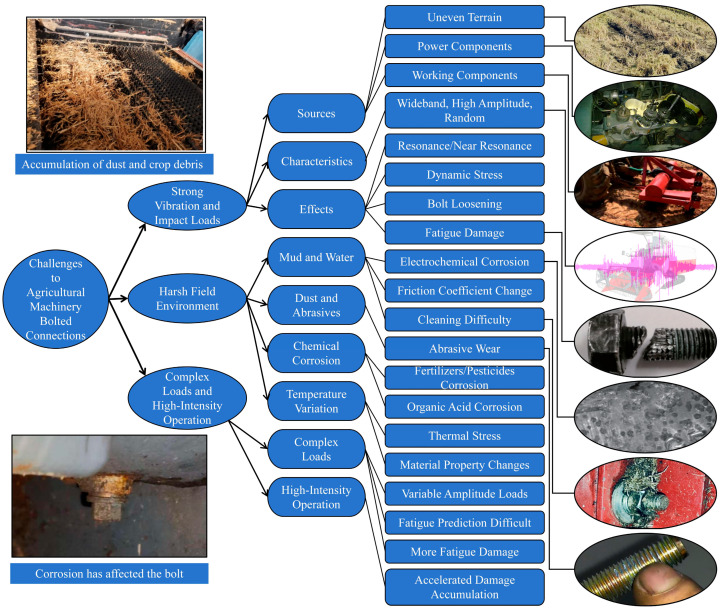
Impacts of specialized agricultural machinery operating conditions and environments on bolted connection reliability.

The operational environment and working conditions associated with agricultural machinery are substantially more complex, and the terrain configuration and complex operating conditions cause high instantaneous torques that exceed the maximum permissible level for bolted joints, mechanical power transmissions, and other machine elements. Some examples in practice are cases when the working bodies of auxiliary machines encounter anthills, stones, or tree branches. At that moment, the resulting torques on the working parts of agricultural working and attached machines are much higher, which can lead to damage to individual elements [66,67,68]. The specific impacts of these specialized operational conditions and environments on bolted connection reliability are illustrated in Figure 5.

### 3.1. Severe Challenge Posed by Intense Vibration and Impact Loads

During field travel and operation, agricultural machinery inevitably experiences intense vibration and impacts originating from multiple sources [69]. Firstly, uneven terrain, ditches, and obstacles like stones subject the chassis and running gear to continuous random vibrations and transient shock loads [70,71]. Secondly, the operation of power components, such as engines and drivetrains, generates intrinsic periodic or non-periodic vibrational excitation [72,73]. Furthermore, the interaction of various working implements with soil, crops, or other materials produces severe operational impacts and reaction forces [74,75]. These vibration and impact loads are typically characterized by wide frequency bands, high amplitudes, and strong stochasticity [76,77]. Such conditions readily excite resonance or near-resonance phenomena within the machinery structure and its bolted connection systems [78], leading to dynamic stresses at connection points far exceeding those anticipated under static loading conditions [79]. Critically, this dynamic environment provides a continuous external energy input conducive to bolt self-loosening, significantly increasing the risk of bolts rotating loose due to vibration-induced micro-slip [80,81]. Simultaneously, it drastically accelerates the fatigue damage accumulation process within the bolted connections [82], thereby posing the most direct and severe challenge to their long-term reliability [83,84].

### 3.2. Accelerated Degradation Due to Harsh Field Environments

Beyond dynamic loads, agricultural machinery must operate within exceptionally harsh physical and chemical environments, imposing significant detrimental effects on the materials and performance of bolted connections [85]. The specific impacts of these harsh field conditions on bolted connections include the following:

(1) Mud, Water, and Humidity: Field operations frequently involve exposure to mud splashes and high humidity [86]. The presence of moisture directly accelerates electrochemical corrosion of bolts and clamped components [87,88], leading to surface rusting, reduced material strength, and impaired thread engagement. Furthermore, moisture infiltration into thread gaps can alter friction coefficients, impacting the accurate application and retention of preload, while adhered mud complicates cleaning and inspection procedures [89,90].
(2) Dust and Abrasive Particles: Significant quantities of dust and fine soil particles are generated during field activities, particularly tillage and harvesting [91]. These hard particles can infiltrate contact interfaces (e.g., thread pairs, under-head bearing surfaces), acting as abrasive media. During relative micro-motion between components, this leads to severe abrasive wear, causing surface damage, diminished connection precision, and potentially inducing stress concentration [92,93,94].
(3) Chemical Corrosion: Agrochemicals widely used in production (fertilizers, pesticides, herbicides) [95], along with organic acids from decomposing crop residues, possess corrosive properties [96]. When these substances adhere to surfaces or dissolve in ambient moisture, they initiate chemical reactions with bolt materials, accelerating corrosive processes [97]. This effect is particularly pronounced under conditions of high temperature and humidity [98], ultimately weakening bolt strength and impairing preload retention capability.
(4) Temperature Variations: Agricultural operating zones often experience substantial diurnal and seasonal temperature fluctuations [99]. Differential thermal expansion coefficients between connected materials can induce thermal stresses within the joint, affecting preload levels [100]. Additionally, exposure to extreme temperatures (both high and low) can alter the mechanical properties of materials, thereby influencing connection reliability [101].

These environmental factors frequently coexist and interact synergistically, collectively accelerating the degradation process of bolted connections and significantly curtailing their effective service life [102].

### 3.3. Cumulative Damage Under Complex Loading and High-Intensity Operation

Dissimilar to many industrial systems operating under relatively stable conditions, the load spectra experienced by agricultural machinery are typically characterized by significant complexity and rapid variability [103]. Factors such as machine initiation/cessation, maneuvering (turning), traversing uneven terrain, transitions between different operational modes, variations in soil conditions, and fluctuating crop densities all precipitate rapid and stochastic changes in both the magnitude and direction of loads applied to bolted connections. This variable-amplitude loading characteristic renders fatigue life prediction for bolted connections more challenging and generally exacerbates fatigue damage accumulation compared to constant-amplitude loading scenarios [104]. Furthermore, agricultural production exhibits pronounced seasonality. During peak operational periods (e.g., planting, harvesting), machinery is often subjected to continuous operation for extended durations, frequently exceeding ten hours daily, to meet critical time constraints. This high-intensity, prolonged operational regime dramatically increases the total number of load cycles experienced by bolted connections. Consequently, various forms of damage-including fatigue, wear, and creep accumulate at an accelerated rate, thereby hastening the progression towards loosening and ultimate failure.

### 3.4. Environmental Adaptability Requirements for Monitoring Technologies

The aforementioned severe operational conditions and harsh environments pose significant challenges not only to the inherent reliability of bolted connections but also impose exceptionally high environmental adaptability requirements on the technologies employed for monitoring their status:

(1) Mechanical Robustness: monitoring sensors and their associated mounting must exhibit sufficient structural integrity and fixture stability to endure the intense vibrations and shocks characteristic of agricultural machinery operation, ensuring uninterrupted functionality and stable signal output. (2) Ingress Protection: adequate protection is mandated for sensors and wiring to effectively prevent intrusion by pervasive mud, water, and dust. (3) Corrosion Resistance: resistance to chemical corrosion from agrochemicals necessitates the use of appropriate materials or protective treatments for sensor housings and sensitive elements. (4) Thermal Stability: reliable sensor performance across wide operational temperature ranges is required, often demanding effective temperature compensation mechanisms. (5) Electromagnetic Compatibility (EMC): given the increasing electronic density on modern farm equipment, robust immunity to electromagnetic interference is essential for monitoring system integrity. (6) Long-Term Reliability: sustained operational reliability and measurement accuracy throughout extended service intervals are crucial to minimize maintenance and replacement frequency for the monitoring system itself.

Meeting these demanding specifications often precludes the direct application of monitoring technologies developed for laboratory or standard industrial environments to agricultural machinery. Consequently, targeted design modifications and ruggedization are indispensable, inherently increasing the technical complexity and implementation costs.

## 4. Principles and Classification of Bolt Loosening Detection Technologies

**Figure 6:**
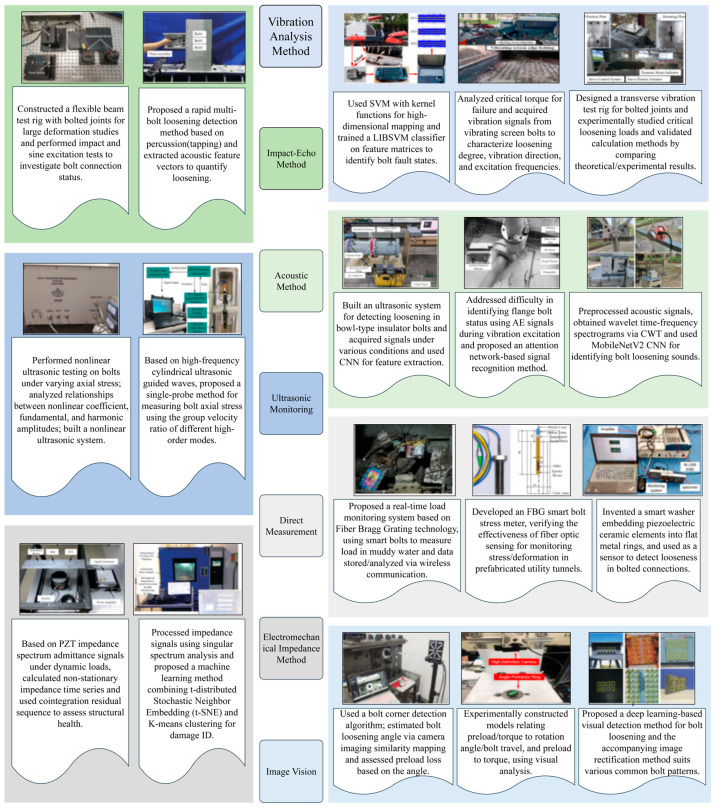
Typical research on bolt monitoring.

To facilitate more effective monitoring and management of agricultural machinery, diverse detection technologies have been developed by researchers. These methodologies function based on distinct physical principles, each exhibiting unique characteristics and specific domains of applicability. This section systematically delineates the principles and features of conventional detection methods, alongside the fundamental operational principles of several predominant modern online monitoring approaches enabled by advanced sensing technologies [105,106,107,108]. Numerous representative studies on bolt monitoring have been conducted [109,110,111,112,113,114,115,116,117,118,119,120,121,122,123,124,125,126], as illustrated in Figure 6.

### 4.1. Principles and Characteristics of Traditional Detection Methods

Traditional detection methods primarily rely on manual operation and experiential judgment, typically conducted during equipment downtime. Despite inherent limitations, these approaches retain applicability in the domain of agricultural machinery, which is often characterized by cost sensitivity and limited maintenance resources.

**Figure 7:**
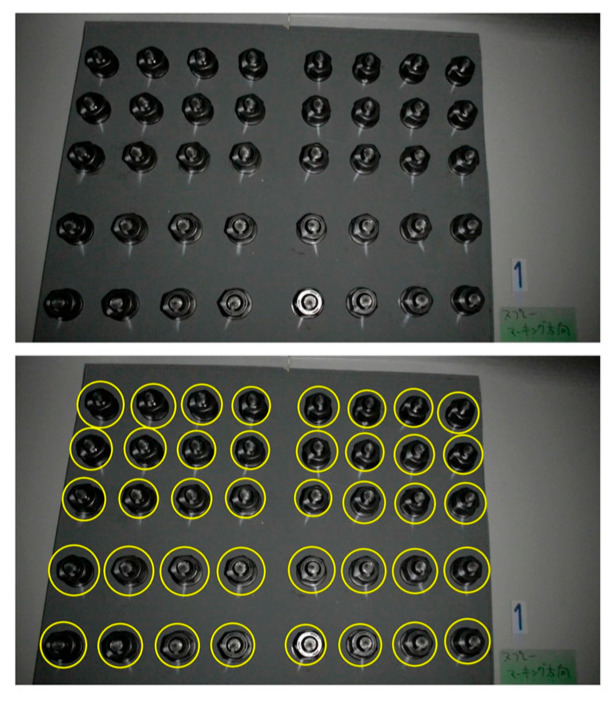
Visual inspection and marking method [127].

Visual Inspection and Marking Methods (Figure 7): Personnel assess potential loosening by observing anomalies at the bolted joint, such as the presence of rust, gaps, or component misalignment. Marking methods involve applying a reference line across the nut and bolt or the clamped component (alternatively using techniques like witness wires or anti-loosening marking paste). Subsequent inspection identifies nut rotation through misalignment of this mark [127,128].

**Figure 8:**
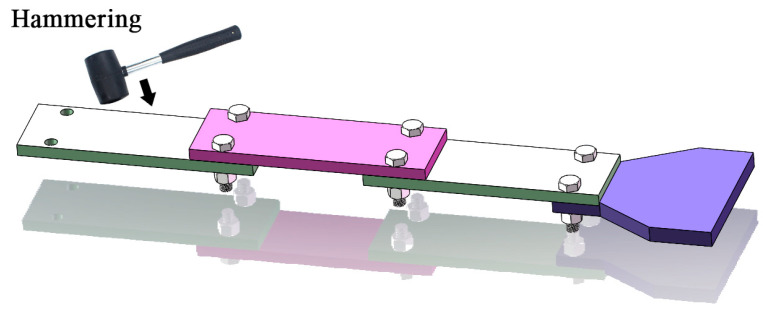
Tapping method for bolt loosening assessment.

Tapping/Listening Methods (Figure 8): Experienced maintenance personnel utilize a small hammer to strike the bolt head or adjacent structure, inferring the approximate tightness state from the resulting acoustic feedback. Some studies also propose judgment based on visual vibrometry following tapping [129,130].

Torque Auditing Methods: These procedures involve applying torque to previously fastened bolts using a torque wrench, typically to assess the angle of rotation required to reach a specified torque value or to determine if further tightening occurs. However, as previously discussed, the relationship between applied torque and resultant preload is subject to significant variability due to factors such as friction coefficients, limiting the accuracy of this method for determining actual clamping force.

### 4.2. Principles of Vibration Signal-Based Detection Technologies

**Figure 9:**
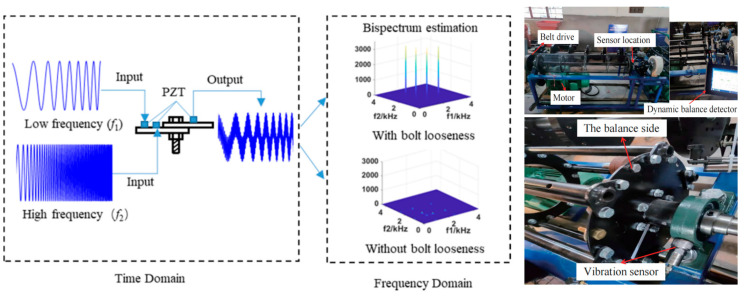
Fundamental principles and process of the vibro-acoustic modulation method for bolt loosening detection [79,143].

Loosening of bolted connections alters the local boundary conditions of a structure, inducing characteristic changes in its vibration signals. It is crucial to acknowledge that agricultural machinery operates in dynamic environments where various components generate inherent vibrations; therefore, the identification of bolt loosening relies on detecting these characteristic changes amidst other potential vibration sources. The underlying principle is illustrated in Figure 9. Commonly analyzed features include the following:

(1) Natural Frequency Shift: bolt loosening typically reduces local structural stiffness, consequently leading to a decrease in natural frequencies, particularly those associated with modes significantly involving the connection [131]. (2) Amplitude or Energy Variation: vibration amplitude or energy levels at specific frequencies (e.g., natural frequencies or excitation frequencies) may exhibit discernible changes correlating with the degree of loosening [132]. (3) Damping Ratio Alteration: loosening can increase friction and energy dissipation at the joint interface, potentially resulting in measurable changes in the structural damping ratio [133]. (4) Nonlinear Characteristics: under vibration, a loosened connection may exhibit nonlinear behaviors such as impacts or changes in contact status. This nonlinearity manifests in the vibration signal through the generation of harmonic components, combination frequencies, or chaotic phenomena [134,135]. (5) Modal Parameter Changes: variations can occur in modal parameters, including alterations in Mode Shapes or a decrease in the Modal Assurance Criterion (MAC) values [136]. (6) Signal Statistical Features: changes in statistical metrics derived from the vibration signal, such as Root Mean Square (RMS), Kurtosis, and Skewness, can indicate loosening [137].

By analyzing the trends of these feature variations, the loosening status of the bolt can be indirectly inferred [138]. Advanced analytical techniques employed for this purpose also encompass Wavelet Transform [139], Hilbert–Huang Transform (HHT) [140], and machine learning-based pattern recognition approaches [141,142].

### 4.3. The Principles of Acoustic Signal-Based (AE/Ultrasonic) Detection Technologies

Acoustic methods leverage the propagation characteristics of sound waves within solid materials to detect bolt loosening [143]. These techniques are primarily categorized into acoustic emission (AE) and ultrasonic methods.

Acoustic Emission: The fundamental principle of AE lies in the phenomenon where localized damage or deformation events within a material—such as micro-crack propagation, plastic deformation, or frictional sliding—release stored energy in the form of transient elastic waves [144]. During the bolt loosening process, micro-scale frictional slip or impacts occurring at the thread contact surfaces or the interfaces between the bolt head/nut and the clamped components can generate detectable AE signals [145]. High-sensitivity AE sensors mounted on the structure’s surface are employed to capture these faint elastic wave signals [146]. Subsequent analysis focuses on various parameters of the acquired AE signals, including ring-down counts, energy, amplitude, and duration, to infer the occurrence and intensity of loosening activity [147,148]. However, effective signal extraction in high-noise environments presents a significant challenge [149].

Ultrasonic Testing: Ultrasonic detection operates as an active method, utilizing the interaction of high-frequency sound waves with the propagation medium [150]. Its application in bolt loosening detection primarily relies on the following principles:

(1) Axial Force Measurement via Time of Flight (TOF): When subjected to tensile preload, a bolt undergoes minute axial elongation. Concurrently, the altered stress state within the material induces changes in the acoustic wave velocity (acoustoelastic effect) [151,152]. By precisely measuring the round-trip transit time (Time of Flight, TOF) of an ultrasonic pulse propagating along the bolt axis, the actual axial stress or elongation can be determined, enabling the calculation of the preload force [153,154]. A decrease in this calculated preload signifies loosening [155]. (2) Interface Condition Assessment: Bolt loosening reduces the contact pressure at interfaces, modifies the effective contact area, and can even introduce gaps. These changes significantly alter the reflection and transmission characteristics of ultrasonic waves encountering these interfaces [156]. Analyzing variations in the energy, phase, or frequency spectrum of the reflected or transmitted waves allows for the assessment of the tightness state of the connection interface [157]. (3) Ultrasonic Guided Waves (GWs): This approach leverages the sensitivity of guided wave propagation characteristics within the bolt structure (acting as a guided wave) to changes in stress state and boundary conditions, such as loosening. Parameters like modal properties, dispersion characteristics, and wave attenuation are monitored for changes indicative of loosening [158,159], for instance, by tracking variations in the phase velocity of specific guided wave modes [160].

### 4.4. Principles of Technologies Based on Direct Measurement

This category of techniques endeavors to measure physical quantities directly correlated with the bolt’s tightening status using more intrinsic methods [161].

**Figure 10:**
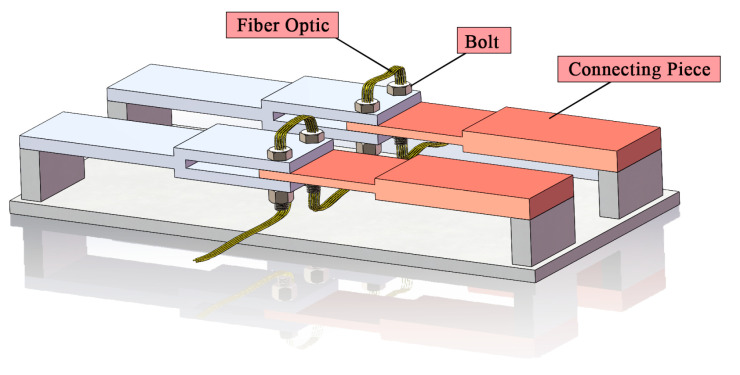
Research on FBG sensing-based smart bolts.

**Figure 11:**
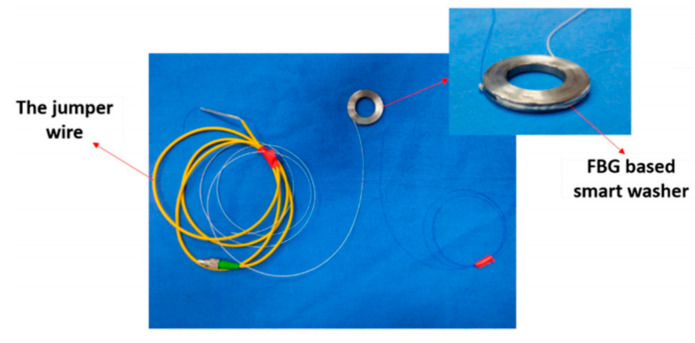
FBG sensing-based smart bolt washer [166].

**Figure 12:**
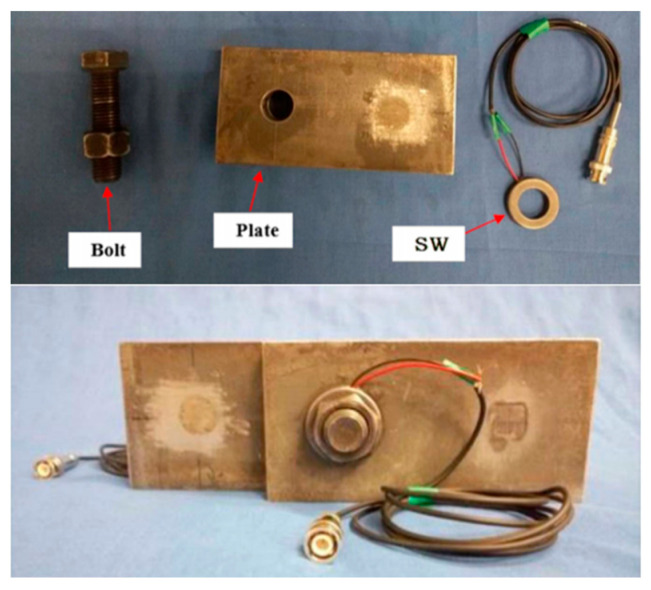
Schematic diagram of smart bolt/washer [121].

Smart Bolts/Washers (Figure 10, Figure 11 and Figure 12): The core principle involves integrating miniaturized sensors directly within the body or onto the surface of the bolt or washer, facilitating the real-time monitoring of key parameters [162]. Common implementations include the following: (1) Strain Gauge-Based: Strain gauges are installed, often in machined grooves on the bolt shank or head, to directly measure the bolt’s axial strain, which is then used to calculate the preload [163]. (2) Piezoelectric Effect-Based: These methods utilize the properties of piezoelectric materials (e.g., PZT ceramics), which generate an electrical charge when subjected to pressure (direct piezoelectric effect) or deform when a voltage is applied (inverse piezoelectric effect). For example, piezoelectric washers can indicate preload by measuring the charge generated under compression [164] or, indirectly, by monitoring changes in their resonant frequency, which is sensitive to the applied force [165]. (3) Fiber Optic Sensing-Based: Fiber optic sensors, such as Fiber Bragg Gratings (FBGs), are embedded within or bonded to the surface of the bolt. Preload is determined by measuring the shift in the grating’s reflected wavelength, which correlates with strain and temperature changes [166]. (4) RFID or Wireless Passive Sensing-Based: These approaches integrate pressure-sensitive elements with Radio-Frequency Identification (RFID) chips [167] or utilize other wireless passive technologies like Surface Acoustic Wave (SAW) sensors [168]. This enables wireless interrogation to retrieve information about the bolt’s preload status [169].

**Figure 13:**
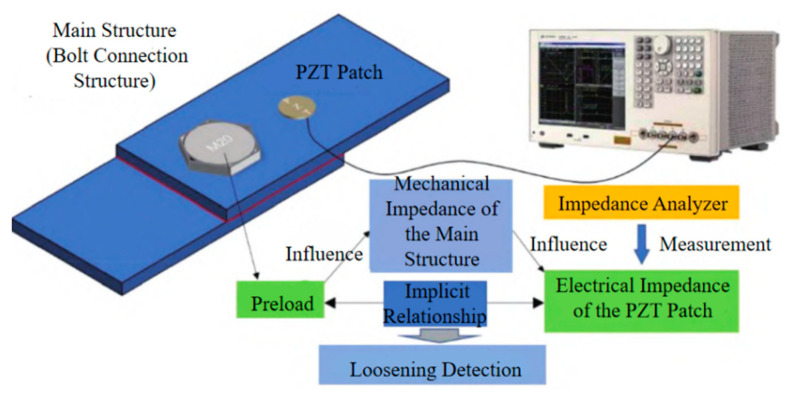
Principle of Electromechanical Impedance (EMI) Method.

Electromechanical Impedance (EMI) Method (Figure 13): This technique leverages the piezoelectric and inverse piezoelectric effects of piezoelectric materials, typically PZT (Lead Zirconate Titanate) patches, for structural health monitoring [170]. A PZT patch is bonded to the monitored structure, often on or near the bolt head or nut surface. By applying a swept-frequency sinusoidal voltage excitation to the PZT patch and simultaneously measuring the resultant electrical current, the patch’s electrical admittance (or impedance) spectrum is obtained. This electrical signature is intrinsically coupled with the mechanical impedance of the host structure to which the PZT is attached [171]. Consequently, any changes in the structure’s physical properties—such as alterations in local stiffness or damping caused by bolt loosening—modify the structure’s mechanical impedance. This modification, in turn, induces measurable changes in the PZT’s electrical admittance/impedance spectrum [172,173]. Comparative analysis between the impedance signature obtained under the current condition and a baseline signature representing the healthy (tightened) state, often quantified using damage indices like Root Mean Square Deviation (RMSD), enables the detection of bolt loosening [174]. Notably, environmental factors, particularly temperature variations, exert a significant influence on the measurement outcomes [175].

### 4.5. Principles of Vision/Image-Based Detection Technologies

Machine vision technology employs cameras to capture images, which are subsequently processed using algorithms to detect bolt loosening [176]. The fundamental principle involves identifying signs of loosening by analyzing temporal changes within images of the bolted connection area [177].

**Figure 14:**
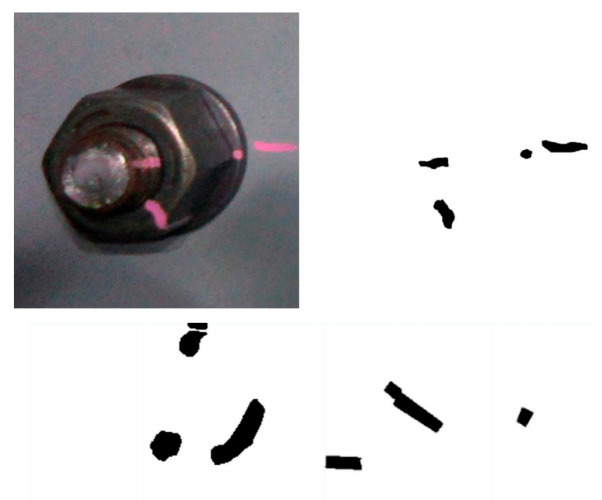
Marker image recognition [127].

Marker-Based Methods (Figure 14): Analogous to traditional marking techniques, this approach involves placing visual markers (e.g., specially designed patterns, QR codes, or simple contrasting lines) spanning the nut and a fixed reference point. Cameras then periodically acquire images. Image processing techniques, such as image registration, feature point tracking, and edge detection algorithms, are utilized to precisely measure the relative displacement or rotational angle of the marker, thereby determining if nut loosening has occurred [178,179].

**Figure 15:**
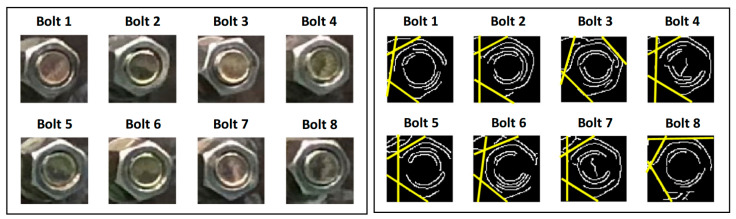
Principle of vision/image-based bolt loosening detection technology [182].

Bolt Geometric Feature-Based Methods (Figure 15): These methods attempt to directly identify inherent geometric features of the bolt head or nut itself (e.g., edges, corners, texture patterns). Loosening is inferred by tracking the changes in the position and orientation of these features across a sequence of images [180]. While obviating the need for pre-installed markers, this approach imposes more stringent requirements on image quality, illumination consistency, and algorithmic robustness [181].

Deep Learning-Based Methods: Utilizing deep learning models such as Convolutional Neural Networks (CNNs) [183], these approaches directly learn discriminative visual features associated with loosening from images of the bolted connection area, enabling end-to-end status classification or detection [184]. While potentially offering enhanced robustness against factors like variations in illumination and partial occlusions, effective training necessitates substantial volumes of labeled data [185,186].

## 5. Application and Limitation Analysis of Detection Technologies in Agricultural Machinery

Despite ongoing advancements in bolt loosening detection technologies at theoretical and laboratory stages, their effective translation into reliably functioning solutions within the complex and variable operational environment of agricultural machinery represents a primary challenge [187,188]. Compared to traditional maintenance, modern sensor-based online monitoring technologies are theoretically better suited for modern agriculture, offering real-time, quantitative, and automated assessment of machinery status [189,190,191]. However, the practical application of these advanced techniques in agricultural machinery remains in its early stages, largely confined to laboratory settings and small-scale trials, with few successful commercial implementations reported [192].

Vibration signal analysis has been explored for monitoring bolt tightness [193,194], typically involving analysis of vibration signals acquired via accelerometers on structural components. However, the complex, high-noise vibration environment inherent to agricultural machinery results in low signal-to-noise ratios (SNRs) [195,196], making practical application difficult, especially as many studies utilize simplified models or simulated conditions [197,198]. Subtle signatures of bolt loosening are easily obscured by background noise. Ultrasonic time-of-flight (TOF) techniques for axial force measurement offer precise preload quantification, proven in high-precision industrial sectors [199], and hold theoretical potential for critical agricultural bolt monitoring [200]. Nevertheless, the agricultural environment (mud, dust, vibration) significantly hinders the stable acoustic coupling required for reliable ultrasonic transducer performance, limiting practical deployment [201,202]. Smart bolts/washers integrating sensors for direct/indirect preload measurement exist primarily as prototypes. Key challenges impeding agricultural application include ensuring long-term sensor reliability and durability in harsh environments, alongside practical difficulties related to power supply and robust wireless data transmission [203,204,205]. Image processing methods monitor marker displacement or nut rotation angles to detect loosening. However, the sensitivity of vision-based approaches to variable illumination, surface fouling, occlusions, and vibration-induced image blur restricts their utility in complex field environments [206,207].

In summary, the principal challenges associated with various modern detection methods are as follows: Vibration analysis for bolt loosening is significantly hampered by the harsh signal environment characteristic of agricultural machinery, leading to low signal-to-noise ratios, difficulties in robust feature extraction, complexity in establishing reliable diagnostic models, and a high susceptibility to misdiagnosis. Acoustic emission (AE) techniques, while sensitive to micro-movements, exhibit poor noise immunity. Direct measurement approaches, encompassing smart bolts and impedance methods, face primary constraints related to environmental durability and the provision of sustained power. Finally, vision-based methods are limited by their sensitivity to environmental variables (such as light and contamination), coupled with the relative complexities inherent in system deployment and subsequent data processing.

## 6. Conclusion and Prospects

### 6.1. Summary of Main Conclusions

Bolted connections play a crucial role in ensuring the reliable operation of agricultural machinery. Among various potential failure modes, loosening is a frequently observed and critical concern. Harsh operational conditions can substantially increase loosening risks, thereby potentially compromising machinery performance and safety. Traditional manual maintenance strategies are demonstrably inadequate for meeting the high reliability and predictive maintenance demands of modern agriculture, suffering from low efficiency, inherent time lags, and an inability to quantitatively assess preload. Consequently, the adoption of advanced monitoring technologies is urgently required. While modern sensor-based technologies (including vibration and acoustic methods) offer potential for online monitoring, their practical application within the agricultural domain remains limited. Significant bottlenecks persist, including poor environmental adaptability, high implementation costs, complex signal processing requirements, and challenges related to power supply and data transmission. Currently, no single technology presents a universally perfect solution; the selection of an appropriate monitoring approach necessitates a comprehensive evaluation based on the specific application scenario, prevailing environmental conditions, cost constraints, and desired accuracy levels.

### 6.2. Technological Trends and Future Prospects

To address the technical bottlenecks in monitoring bolt loosening in agricultural machinery, future research should focus on developing cost-effective sensing hardware with high durability and low power consumption. This includes, for instance, optimizing packaging processes to enhance long-term reliability in harsh environments characterized by strong vibrations and high dust levels, as well as exploring technologies like vibration energy harvesting to resolve the power consumption challenges of wireless nodes [208]. Building on this foundation, advanced signal processing and intelligent diagnostic algorithms must be integrated [209,210] to manage the complex signals inherent to agricultural machinery operations. Simultaneously, it is crucial to actively explore multi-sensor information fusion strategies to overcome the limitations of individual sensors, and to develop customized monitoring solutions tailored to different machinery types and operational scenarios [211,212], supported by in-depth research into bolt loosening mechanisms. Ultimately, the key to success lies in constructing an intelligent maintenance system that is deeply integrated with an agricultural Internet of Things (IoT) framework. This necessitates research into standardized data interfaces and edge computing strategies for efficient data processing and decision support, thereby driving the technology towards maturity through interdisciplinary cooperation and industry application demonstrations [213,214,215,216].

## Data Availability

The data and the related conclusions presented in this article were all derived from the Web of Science database and “CNKI” (China National Knowledge Infrastructure).
